# Ocular Surface Allostasis—When Homeostasis Is Lost: Challenging Coping Potential, Stress Tolerance, and Resilience

**DOI:** 10.3390/biom13081246

**Published:** 2023-08-14

**Authors:** Gysbert-Botho van Setten

**Affiliations:** 1 St. Eriks Eye Hospital, 171 04 Solna, Sweden; gysbert.van.setten@ki.se; 2 Lab of DOHF and Wound Healing, Division of Eye and Vision, Department of Clinical Neuroscience, Karolinska Institutet, Eugeniavägen 12/Level 6, 171 04 Solna, Sweden

**Keywords:** dry eye disease, homeostasis, allostasis, heterostasis, resilience, coping, stress, apoptosis, therapy, recovery

## Abstract

The loss of ocular surface (OS) homeostasis characterizes the onset of dry eye disease. Resilience defines the ability to withstand this threat, reflecting the ability of the ocular surface to cope with and bounce back after challenging events. The coping capacity of the OS defines the ability to successfully manage cellular stress. Cellular stress, which is central to the outcome of the pathophysiology of dry eye disease, is characterized by intensity, continuity, and receptivity, which lead to the loss of homeostasis, resulting in a phase of autocatalytic dysregulation, an event that is not well-defined. To better define this event, here, we present a model providing a potential approach when homeostasis is challenged and the coping capacities have reached their limits, resulting in the stage of heterostasis, in which the dysregulated cellular stress mechanisms take over, leading to dry eye disease. The main feature of the proposed model is the concept that, prior to the initiation of the events leading to cellular stress, there is a period of intense activation of all available coping mechanisms preventing the imminent dysregulation of ocular surface homeostasis. When the remaining coping mechanisms and resilience potential have been maximally exploited and have, finally, been exceeded, there will be a transition to manifest disease with all the well-known signs and symptoms, with a shift to allostasis, reflecting the establishment of another state of balance. The intention of this review was to show that it is possibly the phase of heterostasis preceding the establishment of allostasis that offers a better chance for therapeutic intervention and optimized recovery. Once allostasis has been established, as a new steady-state of balance at a higher level of constant cell stress and inflammation, treatment may be far more difficult, and the potential for reversal is drastically decreased. Homeostasis, once lost, can possibly not be fully recovered. The processes established during heterostasis and allostasis require different approaches and treatments for their control, indicating that the current treatment options for homeostasis need to be adapted to a more-demanding situation. The loss of homeostasis necessarily implies the establishment of a new balance; here, we refer to such a state as allostasis.

## 1. Introduction

**Figure 1:**
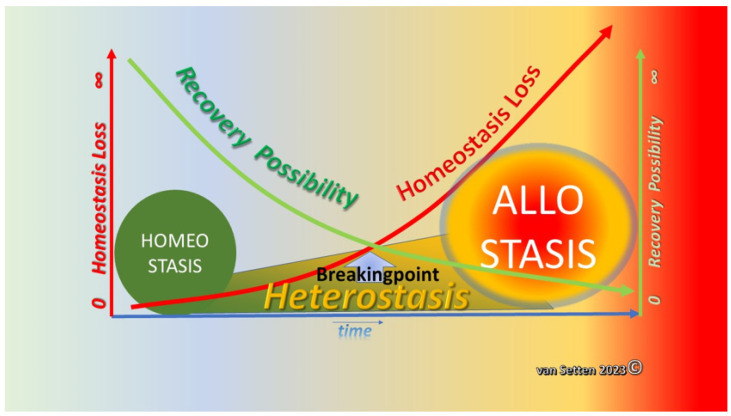
Illustration of showing that the increasing loss of homeostasis (left *Y*-axis) with time will lead to the establishment of a heterostatic phase in which the possibility of recovery decreases constantly (right *Y*-axis). At a breaking point, defining the state when the recovery potential is insufficient to prevent further deterioration and loss of homeostasis, recovery has become impossible. Having passed this breaking point, the recovery potential is swiftly reduced to zero; the final and complete loss of homeostasis is a fact, and allostasis is established as a new balance of cellular survival.

Homeostasis is the concept of balance [1,2], also described as a relatively stable equilibrium between interdependent elements [3]. The alteration of such an environment involves a wide range of molecular events that lead to cellular stress. Stress, as an unspecific reaction of an organism encapsulating the generalized effort to adapt itself to a critical situation [4], has become a respected subject of research. As a whole, it represents the designation of a process stretching a living entities’ comfortable range and homeostasis during exposure to challenges. It describes the first reaction launching the adaptive response to threats to homeostasis. From psychology to biology and the single cell, it affects behaviour, reaction, and survival. It can be a defining parameter of how long a living organism can survive environmental challenges. Naturally, this is dependent on the vulnerability of the organism or cell. Here, resilience comes into the picture, defined in physics as the ability to retake the original form after deformation or in psychology as the ability to endure challenges and to bounce back. A similar concept exists in psychology [5]. The ability to withstand external alteration and deformation is achieved at the cellular level with various coping or defence mechanisms, which also serve to maintain the homeostasis at the ocular surface. Here, homeostasis compromises all compartments of the tissues bordering, in one way or the other, the tear film or the “mare lacrimale” [6]. The vital parameters naturally include the composition of the fluids that eventually become the tear film, as well as their quantity. The tissues engaged include the main lacrimal glands and the accessory lacrimal glands, as well as the lids, the lachrymal pathways, the surface of the cornea, the conjunctiva, and their underlying tissue. In homeostasis, all vital parts coexist in their own harmonic balance and contribute to the wellbeing of the other components. They remain in equilibrium in a volatile balance, which allows reacting to external challenges in a autoregulating way, neither causing excessive inflammation, pain, nor other pathophysiological events. In dry eye disease, a vicious circle [7,8] is involved in such challenges to ocular surface homeostasis. Various systems affecting ocular surface balance can, when dysregulated or activated, contribute to the loss of balance, a model recently outlined in detail at the Dry Eye WorkShop II (DEWS II) [9,10]. A swing towards pathology opens the gate to dysregulation. Under this model, the perpetuation of deterioration leads to dry eye disease. However, following the onset of deterioration leading to the establishment of disease, the current models do not offer applicable phases following the loss of homeostasis. Although adaption and rebalancing to new environments are normal processes [11,12,13,14], the elements of rebalancing or establishing a new balance are less well-investigated [15,16,17,18]. Moreover, at some stage during deterioration and following the loss of homeostasis in ocular surface disease, a new balance may exist to prevent the loss of sight or function. Under such a new balance, the cell/tissue creates an environment with optimized parameters for damage control, implying the possible harnessing of these for treatment. Therefore, here, we propose the following model of dry eye disease in which, following the loss of homeostasis, the phase of heterostasis begins [19] as the precursor to the phase of allostasis [20]. Allostasis means “achieving stability through change” [21], implying that (re-)stabilization to a balance is achieved by adaptive change (Figure 1).

## 2. From Homeostasis to Heterostasis

Heterostasis [19] describes the process by which a new steady-state is achieved, leading to allostasis, a different stage of tissue stability [19]. Heterostasis, as a temporary condition, challenges the balance of the Homeostatic hierarchy of supportive and primary cellular functions [22]. The transition of homeostasis to allostasis results in a phase of regulatory unrest in tissues, responding to environmental stress by using the full coping capacity. Coping capacity is understood as the sum of all availably clinical mechanisms aimed at regaining and re-establishing homeostasis. Heterostasis is a stage of cellular instability in responding to environmental challenges. In this phase, regulatory and autobalancing mechanisms are activated and, eventually, maximized. This can, dependent on the characteristics of the challenge, lead to an alteration and, finally, the exhaustion of all available coping mechanisms, leading to clinically visible ocular surface damage [23]. When the normal mechanisms of defending and coping have been exceeded and homeostasis is lost, a pathophysiological condition is established, heterostasis. As cells, localized at the surface, unlike living organisms, cannot flee from the environment following the concept of “fight-or-flight” [24], they will have to follow the concept of “adopt-or-surrender”. Adaption and the resulting change are the hallmarks of allostasis, a phase in which, by “stability through change”, a new equilibrium is established [25]. It is the activation of the adaptive pathways that occurs when eukaryotic cells are exposed to stress [26] that characterizes allostasis. This condition differs from homeostasis in the current model of dry eye disease (DED) in which treatment focuses on the restoration of the homeostasis of the ocular surface system [27]. This current model is based on the concept that the re-establishment of homeostasis is possible, but does not consider its potential impossibility. 

Accordingly, dry eye disease is a manifest ocular surface disorder and the target for an increasing plethora of treatment regimens [28]. The basic and fundamental difference is that homeostasis and allostasis demonstrate pathology at different levels [29]. This includes assessing the local matrix metalloprotease (MMP) levels such as MMP-9 [30], a group of enzymes known to regulate extrasellar matrix turnover and hyperosmolarity [31,32,33]. This approach, however, considers dry eye pathogenesis as a continuous process, leading necessarily to autocatalytic deterioration, unless this vicious circle is stopped. The variation of the analytic values supporting this model, usually deriving from microlitre quantities and analysed with high-tech precision, is still an issue [34]. This contributes to the ongoing discussion on the representability and difficulties in the validation of measurable biomarkers [35]. Therefore, there is good reason to consider the variation of the parameters to be as important as the established numerical value of the thresholds. Naturally, these thresholds might be relevant, but their value (alone) possibly does not reflect the actual situation correctly. They may serve best to indicate at which level a variation occurs. Such a more-dynamic approach could also help to master the diagnostic challenges of DED mainly concerning the early stages of the disease, where, in the absence of clear clinical signs, enhanced variation or amplitudes such as blinking frequency or osmolarity are easier to identify. In the severe stages of the disease, on the other side, the clinical signs are often very obvious and easier to detect [36], but, at the same time, difficult to reverse. In these advanced stages of DED, marked by the loss of homeostasis, there is often an inherent need for the use of immunomodulators [37,38,39,40]. 

## 3. Heterostasis—Challenging the Coping Capacity, Increasing Cell Stress, and Touching the Limits of Resilience

The initial phases of dry eye disease are marked by absolute or relative lubrication insufficiency, challenging homeostasis. This activates the natural defence mechanisms of the ocular surface such as increased tearing, blinking, etc., which leads to the initiation of the coping mechanisms and resilience [23,41]. Resilience is the ability to bounce back or regain the original form and function, withstanding or recovering from external potential deforming forces found in many aspects of daily life, which has been known for a long time in psychology [42,43], dentistry [44,45], as well in ophthalmology [46,47]. It has been even used to describe the effects of growth factors on corneal re-epithelialization [48]. As dry eye disease imposes a multicausal challenge, the initial clinical effects often are subtle and diagnosis is, especially in the early phases, not easily achieved [9,49]. The lubrication issues of the ocular surface reflect the insufficiency of at least one of its components, i.e., the medium (tears) or the surfaces. Hence, minor, often barely visible surface alterations, such as the anatomical dry eye [50] with topical lubrication challenges, can easily supersede the performance capacity of normal tears and lead to topical desiccation issues, even more when considering the performance profile of suboptimal tears in the elderly. Age alone could alter the lubrication balance at the ocular surface as both tear composition, on the one side, as well as the cellular stress tolerance and adaption capacity deteriorate with age [51,52]. Any lubrication insufficiency is by its nature an imbalance between the needs of the surfaces and the quantitative–qualitative matching capacity of the tear film. Any lubrication insufficiency can have its origin in an anatomical palpebro-corneo-epithelial anomaly such as corneal scars or a real qualitative or quantitative insufficiency such as issues with the lipid or mucin layers [53,54,55,56,57,58,59,60,61]. The hallmark of qualitative tear film insufficiencies is commonly identified by a shorter tear film (TF) break up time [62], but can also reveal itself as a distorted/altered TF break up pattern [63]. The instability of the TF and subjective discomfort are amongst the very early signs of ocular surface challenge [63,64].

**Figure 2:**
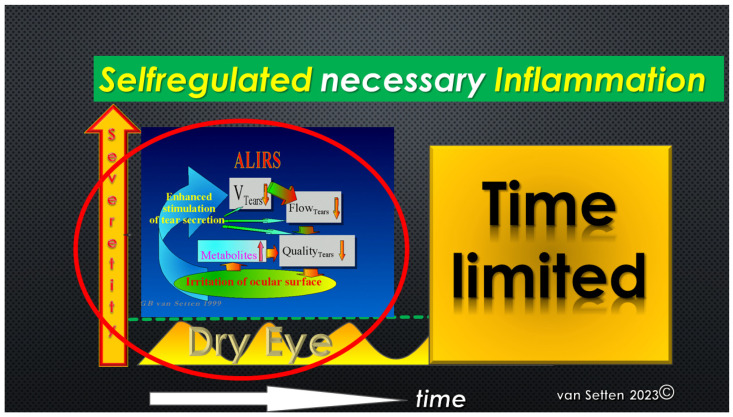
Illustration of repetitive minor inflammation, which leads to temporary and self-limited activation of the acute leucocytic irritative response system (ALIRS). Over time, there is, however, a limitation of how many times or how frequently this system can be triggered before autoregulation fails. The failure of autoregulation marks the end to the ALIRS and the beginning of dysregulation. (van Setten 1999, presented at the founding Meeting of the European Association of Dacryology EAD, October, 13th, Alicante, Spain 2001).

**Figure 3:**
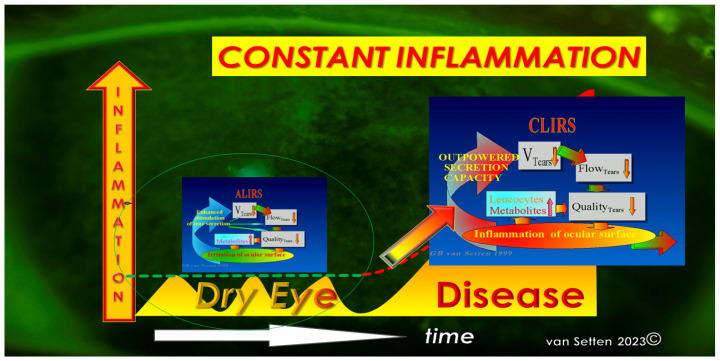
The failure of autoregulation marks the end to the ALIRS and the beginning of dysregulation, i.e., the onset of the chronic leucocytic irritative response system (CLIRS). The activation of the CLIRS includes constant and increasing inflammation, not to mention the attribute of self-limitation. This also marks the onset of ocular surface instability, leading to dry eye as a disease. (van Setten 1999, presented at the founding Meeting of the European Association of Dacryology EAD, October, 13th, Alicante, Spain 2001).

Any early detection or treatment of these initial changes could postpone the onset or slow down the disease’s progress and ameliorate its pathophysiological impact. As recently emphasized [23], the persistence of lubrication insufficiencies could determine the Allostatic Load and the potential of the Homeostatic capacity of the ocular surface to cope with the challenge. Whereas, for temporary lubrication insufficiencies, the acute leucocytic irritative response symptom complex (ALIRS) can temporarily handle inflammatory conditions and retain the coping potential of the ocular surface (Figure 2) [23], things look different when lubrication deficiencies become constant and chronic inflammatory reactions are established (CLIRS) (Figure 3).

The most-common clinical sign of dry eye disease is the occurrence of small micro-lesions in the corneal epithelium. Not only are these evidence of increased friction, but they also constitute a visible sign of corneal damage associated with dry eye disease. As damage always launches repair mechanisms, corneal epithelial fluorescein staining (CFS) is, hence, also a sign of initiated and ongoing wound healing, indicating the presence of a inflammatory response, cellular replication, and the deposition of extracellular matrix [65], as well as altered enzymatic activity, such as increased metalloproteinase activity and plasmin activity [66].

## 4. Ocular Surface Staining—More than Just Colour

**Figure 4:**
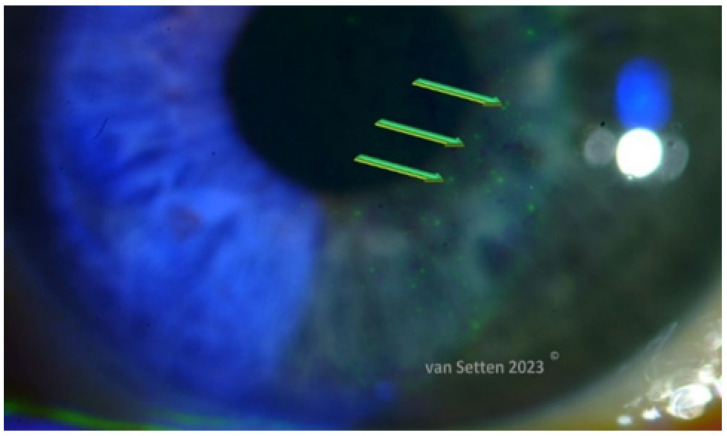
Green arrows indicate localized epithelial defects as stained with fluorescein (van Setten, 2023^©^).

**Figure 5:**
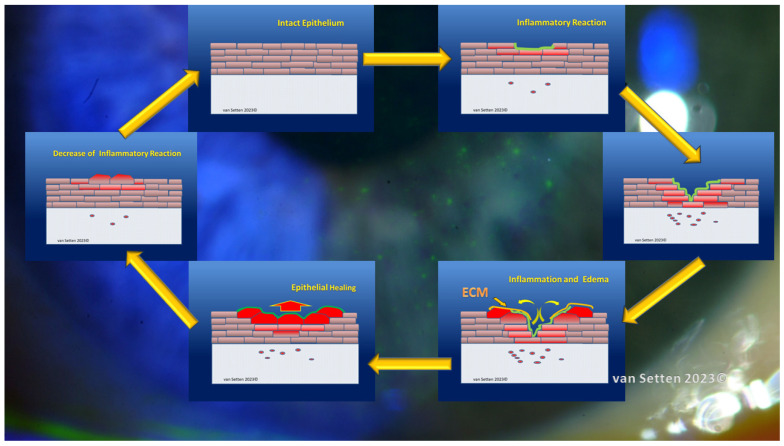
Hot spot theory (HST) (van Setten, unpublished communications 2023) considers that each localized epithelial defect is a corneal micro-wound, leading to a local inflammatory reaction, which intensifies around the lesion and at a depth below it until healing occurs and the epithelium is completely reconstituted (ECM = Extracellular Matrix).

The classification of ocular surface staining according to the Oxford scheme using corneal fluorescein staining (CFS) is performed by counting the spots (Figure 4), which are micro-areas considered as lesions, stained with fluorescein, the number of localized stained spots serving as an indicator of the severity of dry eye disease [49]. Although the location of the staining is known to differ between different areas of the ocular surface, the variation of the staining location is not considered as a characteristic or decisive feature in dry eye pathophysiology. On the other hand, according to hot spot theory (van Setten, unpublished communications, Figure 5), each and every microlesion, resulting in one localized stain, counted as one spot, could be considered as a micro-wound. Any lesion of the cornea or ocular surface leads to local irritation, and an immunological response is launched, which calms down within the normal autoregulation as soon as the epithelial defect has disappeared/healed. Current models of dry eye disease consider ocular surface damage as a constant component, indicating the simultaneous presence of a constant inflammation/inflammatory response. The constant presence of small lesions and their healing suggest the ocular surface to be in a constant stage of wound healing. On the other hand, in situations merely touching the lubrication limits, minor CFS usually means that the lesions are being autoregulated and can swiftly disappear without a trace within less than a day or two. Similarly, the associated inflammation is considered autoregulated and disappears. 

**Figure 6:**
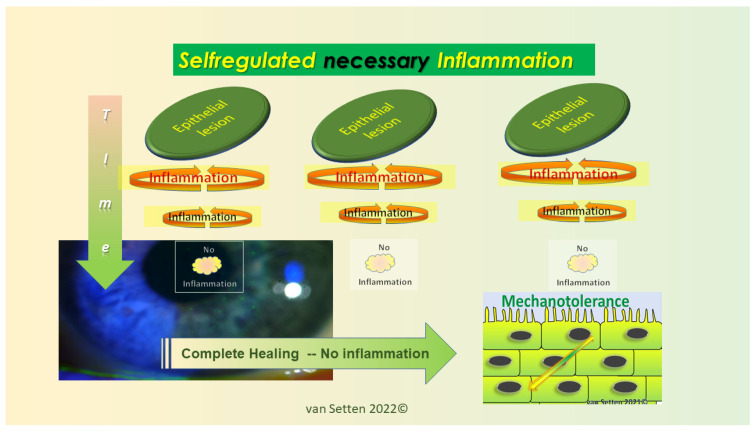
The temporary inflammation under a microlesion, stained well with fluorescein, will (provided the distance to the neighbouring lesion is sufficiently far away) slowly dissolve uneventfully without any remaining inflammation, leading to complete healing of the corneal epithelium. Complete healing indicates complete re-establishment of the original mechanotolerance. (van Setten 2020) [71,72].

**Figure 7:**
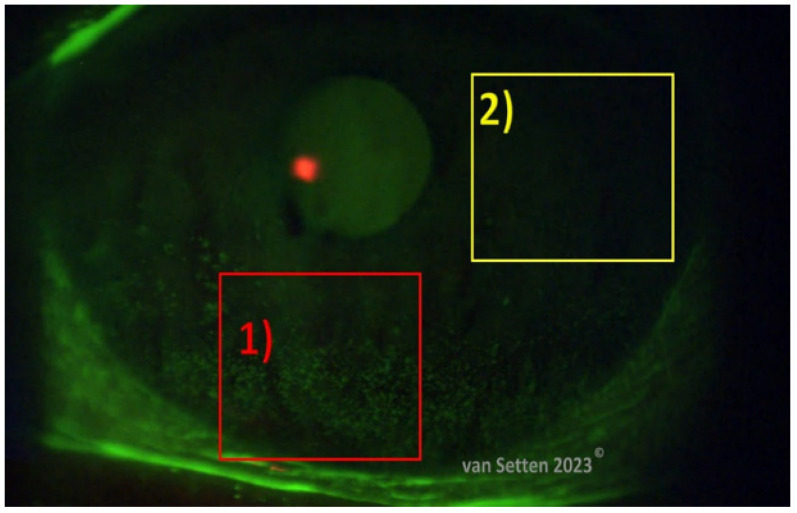
The uneven distribution of fluorescein staining over the cornea. Evidently, there are areas with a high fluorescein clustering index (FCI) (Area 1) and those with a lower FCI in different locations. The likelihood for prolonged and intensified inflammation is considered to be in areas of a high FCI.

Such minor lesions lead to wound healing with the release of inflammatory mediators such as interleukins IL-α and IL-1β [67]. The interaction of these mediators with their receptors results in the onset of inflammation, triggering the onset of ocular surface damage and dry eye flares. Although small lesions heal very quickly in the corneal epithelium, the details of the associated inflammatory reaction have not yet been clearly defined. However, the proximity of lesions does matter. Non-transient, persistent corneal epithelial staining, on the other hand, could indicate the presence of an inflammatory reaction both in the epithelium and in the anterior corneal stroma underneath, which is in the horizontal extension larger than the epithelial lesion itself and could, if the lesions are sufficiently close, easily overlap with the inflammatory zone of the neighbouring epithelial defect. The density of epithelial defects and the local gathering in one area together are possibly more important than the absolute number of dots as used in the classification of epithelial lesions according to Oxford (Figure 6) [68]. It is suggested that, in more-severe stages of dry eye disease, the number of punctate epithelial defects (equal to dots) has reached a critical density in that, in each sector, sufficient epithelial damage has occurred to provide the basis for a continuous inflammatory reaction beyond the surface. Clustering of epithelial defects at a small area (high fluorescein clustering index (FCI)) could determine the intensity (level) of the inflammatory reaction in this area. In spite of the swift healing of each and every individual epithelial lesion, the complete general resolution of any inflammatory reaction as a common inflammation of the area could be much slower. As soon as there is an overlap of inflammatory zones around each epithelial lesion, the pooled (combined) inflammatory reaction underneath the epithelial lesions could contribute to a longer-lasting presence of inflammatory mediators and pro-inflammatory cytokines in the area. Potentially, here, the recently detected G protein-coupled receptor GPR-68 [69] could play a decisive role, as well as the tumour-necrosis-factor (TNF)-stimulated gene 6 (TSG-6) [70]. The swift and efficient decrease of the number of epithelial stainings and an increase in the distance between them seem, hence, to be a prerequisite for efficient therapy of dry eye disease, as shown in Area 2 in Figure 7. 

If, instead, the therapy is not efficient, the number of epithelial defects or staining foci may increase and, with that, the intensity, visualized as a decrease of the distance between the surface alterations, as shown in Area 1 in Figure 7, with a high FCI.

**Figure 8:**
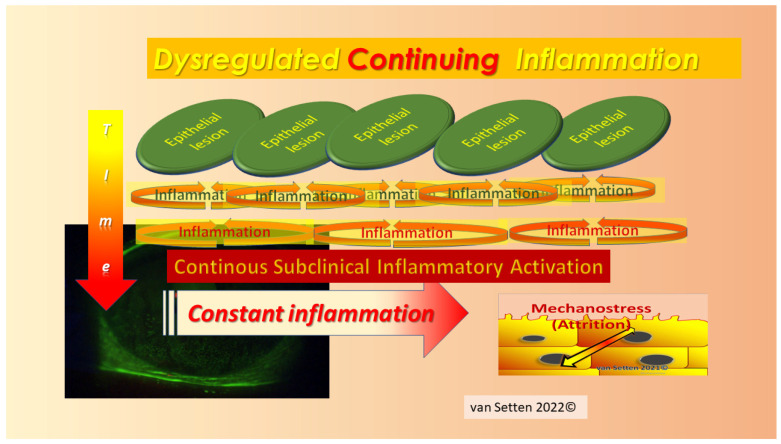
With the increased proximity of the epithelial lesions, i.e., in areas with a high FCI, the initially temporary inflammation under one microlesion could be exacerbated by the coexisting inflammation under other lesions nearby. This could lead to a disruption of the tissue’s ability to dissolve the temporary inflammation. Instead, autocatalytic self-maintenance of an inflammatory stage could establish in the tissue, resulting in constant inflammation with enhanced sensitivity to mechanical stress and attrition (van Setten 2021) [72].

Above a certain FCI of staining, a temporary irritation of the surface with its necessary autoregulated inflammation (ALIRS) could transit to a more chronic, dysregulated, continuous inflammation [71] (CLIRS) (Figure 4 and Figure 8). 

The challenge of homeostasis and the inherent desire of the cell to re-establish its internal balance in order to regain its own functionality and the tissue it is part of activates various coping mechanisms, but also, most importantly, creates cell stress. Cell stress is an essential cellular biological equivalent of stress resulting from lubrication insufficiencies and environmental changes. At present, in dry eye disease, this dominantly contributes to mechano-stimulation such as attrition and friction [71,72], challenging osmotic deviations, as well as hyperosmolarity. 

With the onset of Homeostatic destabilization and imbalance, the cell is pushed outside its comfortable range. Cellular vulnerability and sensitivity increase with the shift of the cell towards the border of its comfortable range, reaching its limits when reaching its maximal coping capacity [41]. Naturally, any cell tries to cope with external challenges by using all its available regulatory mechanisms. The issue with dry eye disease is that the causes are multifactorial and that the cells at the surface have to react simultaneously to a variety of challenges at the same time [73], without having a real chance to recover completely. Whilst in psychology, coping describes dealing successfully with problems or difficult situations [74], coping has been defined on a cellular level as the ability to manage specific external and internal demands that compromise events such as touching, stressing, wounding, or otherwise challenging the resources of the cell/tissue to potentially the maximal extent [23]. Cell stress can be considered as the total challenges exerted by physical, chemical, and biological external conditions affecting the cell. At the ocular surface, this includes, amongst others, mechanical forces and environmental conditions, such as low humidity, temperature, pH changes, radiation, and exposure to toxins. A cell’s ability to cope with stress depends on its ability to recognize changes and to respond appropriately with a plethora of mechanisms, such as the upregulation of pro-apoptotic molecules, the downregulation of anti-apoptotic molecules, and the activation of autophagy, such as activating protective pathways, producing proteins that can protect the cell against the stress, or initiating programmed cell death. Autophagy enhances the cells ability to cope with unfavourable environments [75]. Within *homeostasis*, regulated cell death (RCD) is a necessary and normal process [76]. The processes of uncontrolled, premature, and accidental cell death (an uncontrolled passive process) differ in their regulation and their mediators with a series of molecular mechanisms and signaling pathways [77]. The identification of RCG subgroups such as ferroptosis, necroptosis, and pyroptosis could reflect the cause of cell death [78] and the available coping mechanisms. It is a most likely the relation between the nature of the challenge and the available coping mechanisms that determines whether cells mount a protective, adaptive, or destructive stress response [79]. All depends to a large extent on the nature and duration of the stress, as well as the cell type in question. Cellular resilience, as the sum of all coping mechanisms, describes the ability of a cell to cope with environmental changes [80] that provoke cellular stress. 

## 5. Cellular Stress and Allostatic Load—Driving Forces in the Loss of Homeostasis 

**Figure 9:**
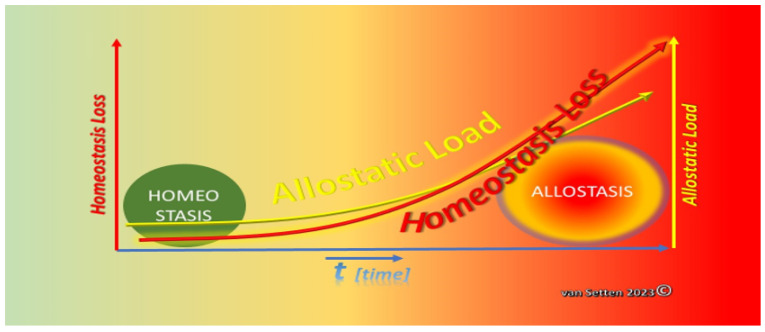
With increased Allostatic Load, there is an increased risk for the loss of homeostasis. If this loss exceeds the potential of coping and a high Allostatic Load persists, adaption leads to a new balance of the system, allostasis. (van Setten 2023©).

Cellular stress requires adaption for survival. Adaption, comprising key features such as resilience, coping, and defence, essentially is the response to the exposure to external stressors constituting as a total the equivalent of the so-called Allostatic Load [25]. This load challenges homeostasis, and similarly, “wear and tear” lead to a pathophysiological disalignment or loss and dys-synchronization of various normally very-fine-tuned processes [81]. Dys-synchronization of cellular mechanisms can lead to severe loss of the efficacy of the processes concerned, potentially jeopardizing the potential of a tissue to appropriately and effectively react to external stressors, i.e., the Allostatic Load. The impact of the Allostatic Load may differ according to the differential expression of disparities [43]. The ability to withstand and cope with the Allostatic Load can be summarized under the term resilience [43]. Long-term stress as part of the Allostatic Load can provoke in neural tissues the loss of plasticity [82]. At the end of the process, when allostasis is established, cells might react differently to the same stimuli as when they were in Homoeostatic conditions. This could result from the exposure to the Allostatic Load, causing an effect of “training” the cells and tissues to satisfy altered external needs [29] and to adapt. The response patterns to the Allostatic Load naturally differ in cells and tissues from the four response patterns of Allostatic Load known from psychology—which, however, all ultimately may lead to chronic disease [82]. Amongst the important similarities, one is the observation that stress leads to changes in the presence or expression of numerous biochemically measurable parameters of intercellular mediators. Furthermore, there is a difference if stress factors, i.e., the Allostatic Load, are temporary or constant. Some Allostatic Loads may essentially not be bad: short-term Allostatic Load can be necessary to keep the system alert and can be essential and without being a major threat to homeostasis, just like the proposed immunological nudging [83]. The potentially changing threat to the system comes with a constant, growing, or always present alternating Allostatic Load, which has the ability to change the cell’s behaviour in a process called adaption [25]. However, in the process of adaption, permanent changes may occur, such as described for the hippocampus with the atrophy of dendrites in response to chronic stress [84,85]. Possibly, there are also links between chronic stress and ageing [86]. Premature senescence of cells and tissues as a result of prolonged Allostatic Load certainly are most likely unreversible. The mechanisms of senescence [87] can contribute to premature ageing and have been associated with various diseases [88,89]. Similarly, normal senescence can impose a reduced tolerability to an otherwise normal Allostatic Load. In essence, over time, the increase of the Allostatic Load would lead to a loss of homeostasis. A constant and/or increasing Allostatic Load can lead to cellular exhaustion, depriving cells and tissues of their full natural coping potential, leading to an initially slow, but constant, later rapidly accelerating loss of homeostasis and the establishment of allostasis (Figure 9).

**Figure 10:**
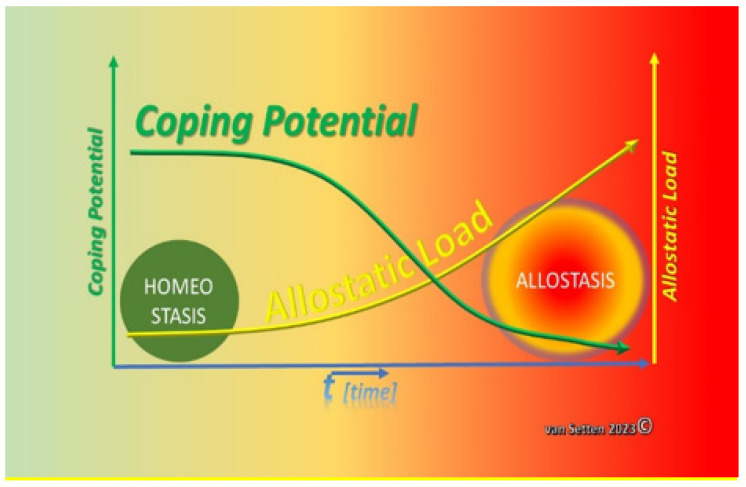
With increased Allostatic Load, the coping potential (necessary to maintain homeostasis) decreases and, finally, will be minimized to inefficiency—giving way to the establishment of a new balance of the system, allostasis (van Setten 2023©).

Adaption, as a response to the Allostatic Load, has. on the other hand, its price, just as in psychology [90], also for the cell. This price is the change of reactions in accordance with the demands of the environment, the only way to survive the sum of all challenges. As pointed out, the characteristics of the chronicity of the environmental challenge are of particular importance, as this contributes to the prevailing stress on the organism [90]. Stress itself is, hence, one of the key factors resulting from the Allostatic Load, challenging the coping potential (Figure 10).

**Figure 11:**
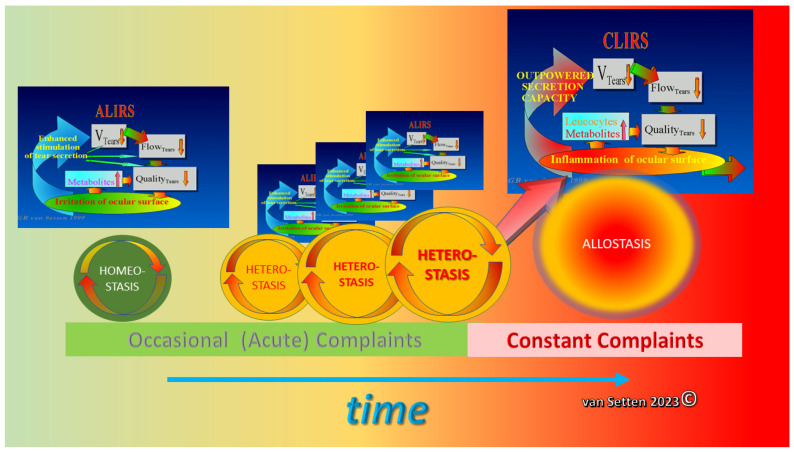
With the increased frequency and intensity of the Allostatic Load, triggering heterostasis, occasional (acute) complaints will become, over time, constant complaints, providing a hallmark of a new balance of the system, with well-established inflammation, allostasis (van Setten 2023©).

As for dry eye disease, the attribute of the reiteration or redundancy of the Allostatic Load without the option to complete recovery can stimulate the transition from homeostasis with only occasional complaints to the state of allostasis with constant complaints (Figure 11). 

In the new, altered phase of stability, the cells have adapted to the prevailing challenges, and allostasis is established. In this new phase of balance, the Allostatic Load causes still may result in constant complaints, but further deterioration has, on the other hand, temporarily stopped. The residual stress tolerance, altered cellular pathways, and coping capacity [91,92] decide the further development, i.e., how long allostasis may be maintained. 

## 6. Stress, Stress Factors, and Stress Tolerance 

Stress tolerance is a prerequisite for cells to persist in varying environments. Eukaryotic cells exposed to stress activate adaptive pathways, allowing them to restore cellular homeostasis [26]. However, cell stress leads to inflammation [93]. Some inflammation, on the other hand, is needed to maintain homeostasis and should be considered as physiological [22], having about a similar invigorating stimulative circuit-maintenance function as described for immunological nudging [83]. Excessive inflammation, on the other hand, has been widely accepted as a driving force in the pathophysiology of dry eye disease [7,8,94,95]. It is less the occurrence of inflammation as such, as more the perpetuation and non-cessation of inflammation that harbours the pathophysiological impact [96,97]. Accordingly, inflammation has become the target of many treatments for DED [98]. The exact mechanism for the regulation of this inflammation, as well as its dynamics and location are, however, still subject to discussion. Here, one of the key actors is nuclear factor kappa B (NF-κB), which is considered a master regulator of inflammation [99]. Inflammatory reactions are, by their nature, not static; they move through tissues to accomplish their goal of defence and tissue reconstruction. It should be kept in mind that that there is a minor, but possibly significant difference between inflammation as such [100] and inflammatory responses in dry eye [101]. As suggested for the retina [102], as well as for the ocular surface, immunological homeostasis seems to be essential. In homeostasis, a plethora of mechanisms are engaged, amongst others corneal innervation and microbiota [103,104]. Once challenged, the maintenance or regain of this balance seems to be dependent on the characteristics of the causing agents or conditioning factors such as the magnitude, i.e., intensity, character, continuity, and recurrence (seasonality) [105]. The sum of these external factors challenging the coping mechanisms identifies the recovery potential and capability of the ocular surface structures [41]. Especially chronic stress, affecting the endoplasmic reticulum, harbours an imminent risk for permanent intracellular changes with links to inflammation [106] and a self-perpetuating inflammatory reaction [95], the very core of the vicious circle of dry eye disease [7,8]. 

Insights into the complexity of the cellular mechanisms have been gained by the use of transcriptomics, measuring the gene expression in cells and tissue. Albeit that bulk and single-cell transcriptomics have each their specific benefits [107,108], both techniques allow looking deeply into the very basic events of cellular physiology. Especially the use of single-cell transcriptomics in neuroscience allows the examination of “cell states” on a molecular level, as well as cell typing [109]. Furthermore, this technique allows gaining insights into the diversity of cellular responses in different cell types. This, in turn, forms the basis of recent models, addressing the issue of how these responses could contribute to the alteration of cells and their plasticity [110]. Next-generation sequencing (NGS) [111,112] allows detailed insights into cell physiology with the analysis of hundreds of genes simultaneously and can be used to distinguish different phenotypes of cells on a molecular level [113]. This was recently shown for the effect of oxidative stress in retinal pigment epithelium [114]. This new technique allows the identification of genes that encode key proteins [115], i.e., genes that are engaged or necessary for cellular physiology and survival. Its capacity to edit the panorama of cellular reactions to desiccation challenge has led to new insights into the pathophysiology of, especially, Sjögren’s Syndrome [116,117,118,119,120,121], although the application of this technique to the cornea and ocular surface is yet very limited [122]. 

**Figure 12:**
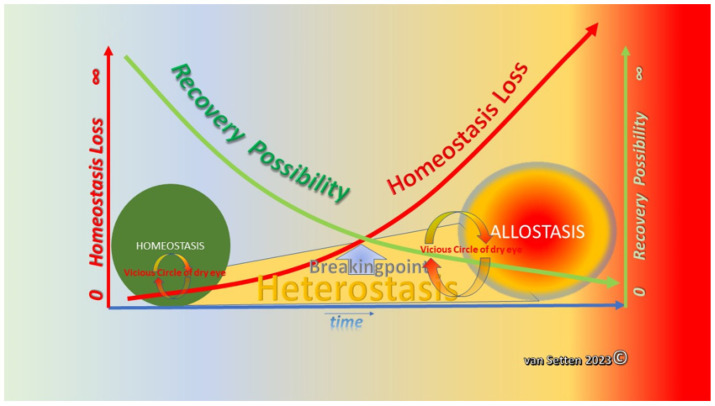
With increased loss of homeostasis, heterostasis sets in, leading the system to a breaking point, identifying the end of a possible restitutio ad integrum. After reaching this point, the possibility for recovery is minimized and, under the continuous threat of the vicious circle, allostasis becomes the new balance of the system (van Setten 2023©).

Regardless of the details of the dysregulation of homeostasis, it is the accelerating imbalance that drives the vicious circle further, supporting increasing heterostasis, eventually finding a new balance within allostasis (Figure 12).

## 7. Hyperosmolarity—More than a Numerical Value

Another stress factor, hyperosmolarity itself has been suggested to be a driving force within the vicious circle [10], albeit that recently, the models on osmokinetics and osmotic variation [71,123] have given more consideration to the importance of osmotic alterations. In osmokinetics, both the amplitude and level around which DVO pivots are suggested to contribute to the intensity of stress provoked [72] and with that, the impact on heterostasis. Within the current model of ocular surface homeostasis, this would suggest an osmotic equilibrium in the tear film hovering around the normal osmolarity with values of around 305 mosmol/L [124,125,126]. This might be the average level without considering the reported variation between samples taken during various time points of the day [32], which was later termed the diurnal variation of osmolarity (DVO) [71]. Such normal variation of osmolarity (<25 mosmol/L per day, unpublished results by the author) apparently falls within the tolerance area of the normal coping envelope of the ocular surface. 

Interestingly enough, osmolarity in lacrimal gland fluid decreases with the flow rate [31]. An excessive flow rate, such as in the initial phases of dry eye disease, can possibly cause inflammation in the lacrimal glands and trigger fibrosis [127]. However, with decreased functionality of the lacrimal glands, tear fluid flow decreases, as evidenced by decreased Schirmer Test results and, with this, probably, also this dilution effect. Such a dilution effect had been shown earlier for cytokines such as epidermal growth factor (EGF) [128]. Alterations in the tear fluid’s composition could enhance the evaporation of a decreased tear film volume and drive osmolarity to challenging levels. At higher levels of average osmolarity, any broader DVO could have a significantly higher stress impact (Allostatic Load factor; see above) as the comfortable range of the cell is exceeded [72,123]. That is, the more the cell experiences osmotic stress, either to the level of osmolarity, the magnitude of DVO, or the frequency of major changes [123], the more sensitive it becomes to fluctuations as the time for recovery becomes shorter and, at a certain point, insufficient. Until then, however, osmolarity provoked stress still leads to temporary inflammatory reactions (ALRIS) that is managed by normal coping mechanisms [71]. Such coping mechanisms are, however, apparently, a time-limited resource. Once recovery options are exceeded, cellular exhaustion might follow, leading to permanent inflammatory reactions. 

The response to osmotic stresses is not only a change of water content and turgor in the cell [129,130,131]. Osmotic variations also change cellular behaviour and can drive the cell to another stage of balance, i.e., allostasis. Osmotic changes have been reported to cause modifications in the cytoskeleton dynamics and molecular crowding in the cytoplasm [132,133]. Elevated external osmotic pressure not only leads to a reduction in the cell volume, but also to an increase in the cell stiffness in different cell types, that is that if a cell is compressed by hyperosmotic stress, it becomes progressively more rigid [133]. This, in turn, would make the cells more vulnerable to the effects of attrition [134], possibly also contributing to increased sensitivity, perceived as discomfort and pain. 

The resilience of the ocular surface is dependent on the coping capacity of the entire system of the surface, in which both the tear fluid and its components [135,136,137], as well as the lids play a decisive role. Coping capacity with osmolarity changes as part of the adaptive stress responses is dependent on the osmo-adaptation time. If the time for adaption or recovery is too short, one feature of the stress response is a downregulation or arrest of the cell cycle [136], which could lead to a further deterioration of the mechanical properties of the thinned epithelium in eyes with dry eye disease. 

The basic cellular mechanisms supporting coping have been suggested [23] to include the secretion of lipids by the Meibomian Glands, the glycocalyx [138] with mucins [61,139,140,141,142], hyaluronic acid production [71,143,144,145,146] and degeneration, epithelial regeneration with multilayering of the epithelium [48], the elasticity of the epithelium [147], and the regulation of neuronal regeneration [148,149,150,151]. 

Stress factors challenging the coping mechanisms are often subtle, allowing the cells to compensate and adjust to the external challenges without exhausting their resources, maintaining their functional integrity and that of the tissue they are a part of. In the earlier model of dry eye disease including temporary acute inflammatory reactions (ALIRS), the time interval for coping challenge was limited, providing the cells the time needed to recover and readjust after a period of challenge. An insufficient time of recovery by repeated exposure to significant external cell stimuli could lead to the final exhaustion of the cell and the functional disability of the tissue it is part of. Part of the cells’ own re-adjustment reactions can include the release of mediators to the environment in response of the cells’ exposure to stress, resulting in inflammatory reactions, leading to additional changes in the environment of the cell [152,153,154]. Here, for example, the accumulation of inflammatory or pre-inflammatory mediators could lead to inflammatory preconditioning. There is an elevated potential of immediate inflammatory reactions upon additional stimuli, pre-dispositioning the cell or the tissue to potentially overwhelming inflammatory reactions, causing at the end the loss of the Homeostatic balance in the tissue concerned. Even after the cessation of the originally stress-provoking environmental factors, the time for completely solving the inflammatory alterations in the environment of the cell could require more time than the recovery of the cell itself. Any premature re-exposure of the cell to stress under these conditions could have for the cell a far higher detrimental effect and cause different cellular reactions than at the initial exposure when the original unused and fresh coping mechanisms were being utilised by the cell to the full extent. This is the essence of the dynamic models of dry eye disease addressing attrition [72] and osmolarity, i.e., osmokinetics [71,123]. In hyper-osmotic conditions, the endoplasmatic reticulum (ER) of corneal epithelial cells can be disturbed, resulting in pro-inflammatory signaling [155]. This emphasizes the role of the ER in the cellular response to stress. Similar to osmotic stress, also mechanical forces can provoke some of the epithelium reactions at the level of the ER. Overstretching of lung tissue inducing epithelial ER Ca^2+^ release has been shown to activate PERK-specific ER stress signaling [156]. Increased friction in dry eye disease [157], enhanced attrition [133], and the thinning of the epithelium [158] could indeed contribute to a mechanically provoked PERK activation. Interestingly, epithelial thinning was considered to be even a result of mechanical friction [158]. On the other hand, also, enhanced epithelial surface irregularities in dry eye disease [159] could contribute to the uneven distribution of mechanical forces applied on the surface, for example by the lids. 

## 8. Inflammation—From Necessary Asset to Pathological Threat

In all common models of dry eye disease, inflammation plays a key role. The difference is, however, the identification of the specific nature of the inflammation. When homeostasis is challenged, i.e., in the early phase of homeostasis, minor, repeated Homeostatic challenges and destabilization can trigger autoregulated inflammation, as part of the innate corneal immunity [160,161]. Here, it can trigger temporary inflammation in the cornea, forming the basis for inflammatory flares [162]. Inflammatory reactions of the ocular surface have been already suggested to negatively affect even the conjunctival epithelium [163]. Inflammation as an essential part in the pathophysiology of dry eye disease has been reviewed recently [9,164] and cannot be discussed expressively here. 

Inflammation itself is a necessity part of tissue self-maintenance [82] and should be handled with diligence when therapy is applied. It contributes to the activation of regenerative cycle mechanisms, which lead to the substitution of damaged or incapacitated tissue with new vital tissues with full potential. By its nature, such inflammation occurs in a certain interval or with a periodical redundancy in order to keep tissues in good condition [165]. Eliminating dead and dysfunctional, apoptotic, and necrotic cells by apoptotic processes [166,167,168] has immunological consequences. Such apoptotic clearance is essential in tissue homeostasis [169]. Accordingly, in normal systems, well-controlled apoptotic mechanisms even have an anti-inflammatory effect [170]. Possibly, these mechanisms face an overload due to exaggerated cell loss and shedding in severe ocular surface damage such as in severe dry eye. The normal defence system as part of the coping potential, operating with the principles of immunological nudging, keep the inflammation-triggering immunological systems in the appropriate states of vigilance, handling the elimination of dead and dysfunctional cells [166,167]. Although it can come with immunological consequences [168,171,172], the clearance of apoptotic cells is critical for maintaining normal immunity [173]. In this context, for dry eye disease, mitogen-activated protein kinase (MAPK) activation is considered a core mechanism according to DEWS II [10] and, hence, is part of the processes engaged in the coping mechanisms. Hyper-osmolar stress leads to the activation of MAPKs, as well as nuclear factor (NF)-κB [174]. This also offers a new window of therapeutic accessibility. As shown in yeast, the exposure to high osmolarity can induce via MAP kinase the synthesis of glycerol and, thus, increase the internal osmolarity [175]. Similarly, it was shown that the MAP kinase system can be activated by using hyperosmolar medium covering the epithelial cells [174]. 

MAPK regulates various cellular activities including proliferation, differentiation, apoptosis or survival, inflammation, and innate immunity [176]. Accordingly, the inhibition of the p38-MAPK pathway has been suggested in the dry eye management associated with Sjögren’s syndrome [177]. The activation of the MAPK signaling pathway in an experimental dry eye model has already earlier been the target for the development of new treatment options [178], all the more as MAPK signaling pathway activation can result in NLRP3 inflammasome formation [179]. Inflammasomes control parts of inflammation [180] and are multiprotein signaling platforms that control the inflammatory response [180]. Inflammasomes play an important role in the progression of fibrosis as innate immune receptors. There are four main members of the inflammasomes, such as NOD-like receptor protein 1 (NLRP1), NOD-like receptor protein 3 (NLRP3), NOD-like receptor C4 (NLRC4), and absent in melanoma 2 (AIM2), among which the NLRP3 inflammasome is the most-studied [181]. As for the pathophysiology of dry eye disease, it is not yet known which NLRP is of most importance in driving the inflammation during the progress of the disease. 

The challenge of homeostasis results in ongoing inflammatory reactions during the entire progress of DED. As part of the coping system during inflammation, IL-37 suppresses the expression of several pro-inflammatory cytokine in favour of the expression of anti-inflammatory proteins. This can be achieved by the regulation of macrophage polarization, lipid metabolism, inflammasome function, TSLP synthesis, and miRNA function. Extracellularly, IL-37 forms a complex with IL-18Rα and IL-1R8 (formerly TIR8 or SIGIRR), which transduces anti-inflammatory signals by the suppression of the NF-κB and MAPK and the activation of the Mer-PTEN-DOK pathways [182]. In the immune system, autophagy is not only an essential part of tissue remodelling [183], but also a cell response to stress [184]. In the coping mechanisms of the ocular surface, autophagy plays a key role as its stimulation has been shown to mitigate the stress-induced inflammation in human corneal cells [185]. Specifically, for trehalose, this is due to p38MAPK inhibition, but not NF-κB. Autophagy in general is very closely linked to inflammation [186,187,188,189] as the crosstalk between autophagy and inflammatory signaling pathways is essential to balance defence and homeostasis [190]. Furthermore, autophagy is engaged in the regulation of inflammasomes [191] and limits their activation [192].

## 9. Summary—Allostasis as a Situational Adaption and a Chance for Survival

**Figure 13:**
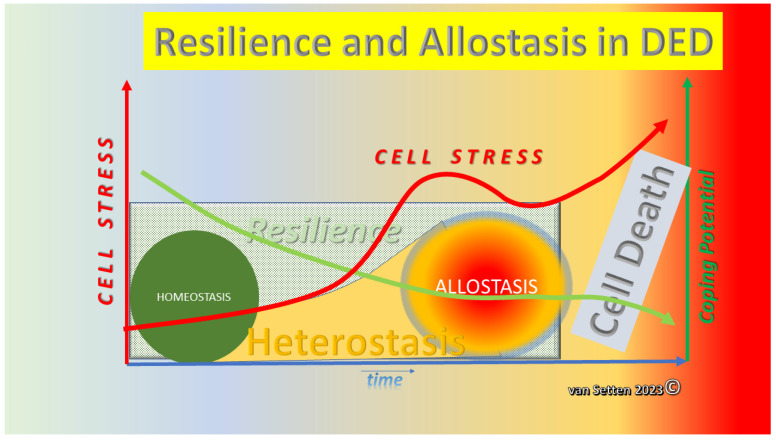
With increased cell stress, resulting in a decrease of resilience, homeostasis is lost and heterostasis sets in, leading the system to a new phase of adaption, allostasis. Continued exposure to stress and a high Allostatic Load leads, finally, to the total exhaustion of an already minimized coping potential and, eventually, to the death of cells and tissues (van Setten 2023©).

Dry eye disease implies a chronic challenge to the homeostasis of the ocular surface. Homeostasis, as a model of physiological balance, includes a variety of separate regulative mechanisms and their components, each establishing a functional, autoregulative unit. In the current model of homeostasis, as state of equilibrium, each parameter is supposed to maintain a certain value [193], this supporting the functionality of the system. This includes the resistance to external stress factors and implies a certain, balanced fluctuation of self-adjustment within the system. This allows the ocular surface to survive and cope with the events outlined in the vicious circle of dry eye disease [7,8]. However, nominal normal values hardly can reflect a dynamic system and its autoregulative capacity. The Allostatic Load requires a well-adjusted system of coping mechanisms to withstand a shift to an unstable situation of Heterostatic imbalance, which can ultimately lead to the establishment of an Allostatic phase, allostasis. Allostasis is the situation after adaption to challenges with some modifications of the earlier state of balance. Allostasis offers “stability through change” [194], emphasizing the need to adapt. Therefore, the general and individual coping capacity of the ocular surface and its regulatory potential is of the highest pathophysiological relevance. Especially during the early phases of ocular surface disease, the capacity of coping, an essential part of resilience, is of major importance as the issue is the activation of the vicious circle of DED. Prior to the entering of any vicious circle, there is a time of intense activation of all available coping mechanisms dealing with the imminent dysregulation of the ocular surface’s homeostasis. This phase of coping with the desiccation challenge offers both a chance for early therapeutic intervention and early diagnosis. Helping the ocular surface avoid permanently losing its balance by recognizing, supporting, and enhancing possibly present (but insufficiently effective) compensatory mechanisms could possibly reverse or slow down the velocity of deterioration. Neither resilience, nor the coping capacity of the ocular surface are unlimited (Figure 13).

Resilience as the ability to cope is dependent on the magnitude of the stress factors and can, at the end, decrease to zero when stress leads to a loss of cellular functionality and, later, cellular and tissue integrity—not allowing any further chance for recovery. At the final stage of continuous deterioration of DED, then, when even the impaired balances characterizing the Allostatic phase begin to fail, the definitive loss of the functional integrity of the ocular surface is imminent. Then, dry eye disease has indeed become a real threat to sight. Here, in the presence of therapy-resistant ocular surface desiccation, surgical procedures become the last resort [195,196]. Better models, reflecting more accurately the dynamics of the pathophysiology, are needed to prevent this. The reconsideration of dry eye disease as more than a challenge and the loss of homeostasis, but as volatile stages of lost homeostasis and permanently altered ocular surface imbalance the without possibility to return could be an important step on this way. 

## 10. Conclusions

Treating lubrication insufficiencies in time, i.e., in the early initial phases, could, possibly, significantly extend the time of coping, postponing permanent changes in the cellular mechanism as a result of desiccation stress, alleviating subjective discomfort and the decrease of performance abilities. When, during the time of ongoing DED, the optimal treatment windows during heterostasis have passed and allostasis has replaced homeostasis, any treatment will need to address even more immunological mechanisms, but with significantly decreased hope to potentially restore the primary condition with its Homeostatic autoregulatory mechanisms. Finally, as the prevalence of DED does increase with age, this also alters the presence and regulation of inflammation in human tissues. Hence, the coping mechanisms and the ability to handle inflammation in the context of dry eye disease obviously could differ between younger and older people. Eventually, the phenomenon of “inflammaging” [197] could also be considered as a predisposition to enter a vicious circle of the ocular surface, leading to dry eye disease. In these conditions, the coincidental higher rate of ectropia or other apposition errors of the lid might be more important for the development of dry eye disease than has been thus far presumed. The identification of such Allostatic Load factors could offer an important step to avoid the loss of homeostasis and the transition to allostasis.

## Data Availability

Not applicable.
